# Accelerated long-term forgetting reveals everyday memory deficits in early-stage multiple sclerosis

**DOI:** 10.1007/s00415-024-12359-4

**Published:** 2024-04-08

**Authors:** J. Stalter, K. Pars, K. Witt

**Affiliations:** 1 Department of Neurology, School of Medicine and Health Sciences, Carl von Ossietzky Universität Oldenburg, Oldenburg, Germany; 2 University Clinic for Neurology, Evangelical Hospital, Oldenburg, Germany; 3 Center of Neurosensory Sciences, Carl von Ossietzky Universität Oldenburg, Oldenburg, Germany

## Abstract

**Background:**

Patients with multiple sclerosis (MS) patients report subjective memory impairment (SMI) escaping routine neuropsychological testing. Accelerated long-term forgetting (ALF) refers to above average loss of information over an extended period of time (e.g., 7 days). This study investigates ALF in mildly affected MS patients and relates long-term memory performance to SMI.

**Methods:**

This prospective study included 30 patients with early MS (mean EDSS ± SD = 1.1 ± 0.9) and 30 healthy controls (HC) matched for age and education. Participants underwent ALF testing [word list (RAVLT), geometric figure (RCF), logical memory (WMS)] at three time points (baseline, 30 min, 7 days). Cognition (Montreal Cognitive Assessment), depression, SMI and fatigue were assessed. The primary outcome (PO) was defined as the quotient of the 7-day score and the 30-min memory score for the verbal (RAVLT, WMS) and figural (RCF) memory tests. The study was approved by the local ethics committee and is registered in the German Register of Clinical Studies (DRKS00025791).

**Results:**

MS patients showed impairments in PO_RAVLT_ (MS 0.66 ± 0.13 vs HC 0.82 ± 0.16; *p* < 0.001), whereas PO_WMS_ (MS 0.88 ± 0.15 vs HC 1.01 ± 0.12; *p* = 0.02) showed only a tendency. Regression analysis revealed significant associations for PO_RAVLT_ and fatigue (*p* = 0.034), and PO_RAVLT_ and SMI (*p* = 0.01) in patients but not in HC.

**Conclusion:**

The ALF test quantifies SMI in MS-patients. With fatigue as a relevant associated factor, this fills the gap in objectifying SMI in MS for diagnostic purposes.

## Introduction

Multiple sclerosis (MS) is a chronic autoimmune-inflammatory neurological disease that leads to cognitive impairment in 45–65% of patients [1]. Subjective memory impairment (SMI) can be considered as first cognitive symptom in MS that affects the lives of patients, especially in daily activities such as social and occupational status [2, 3]. SMI is present, when patients report an impaired memory in everyday life, while performance on objective standard neuropsychological test is unremarkable [4]. In other words, a subjectively reported impairment that corresponds with objective test results should not be classified as SMI according to the definition of Abdulrab and Heun [5]. Accelerated long-term forgetting (ALF) is evident, when new information can be encoded and recalled normally on standard tests, but recall is impaired when tested after longer periods than usually assessed (e.g., 7 days), thus ALF escapes standard memory testing [6]. Even though, the research field ALF originated from epilepsy, this condition has been shown in various other diseases, such as stroke, limbic encephalitis, Parkinson’s disease and Alzheimer’s disease [7–11]. Another point that has to be highlighted are the methodological issues, e.g., test material or delay period, that come with the investigation of ALF since there is no broadly accepted concept to test for it yet. To overcome these issues, we applied the recommendations by Elliot et al. 2014 to ensure a valid testing, including among others, strict matching of patient and control groups, use of verbal and non-verbal test material avoidance of rehearsal and matching of initial learning [12].

The fact that SMI cannot be clinically objectified may reduce awareness and acceptance and therefore, delay the initiation and the development of treatment options. In this prospective exploratory study, we investigated whether ALF is present in early-stage MS and whether it is associated with SMI. Furthermore, the relationship of ALF and SMI to fatigue, depression, and memory questionnaires will also be investigated.

## Methods

### Participants

Inclusion criteria were the German language as mother tongue and a definite diagnosis of RR-MS according to the revised McDonald criteria [13]. Exclusion criteria were medication with effects on memory, a neurological diagnosis other than MS or an EDSS score > 3. Since cortisol can have effects on cognitive functions, we defined a treatment with cortisol due to an exacerbation in the last 6 weeks as an additional exclusion criterion. Healthy controls (HC) had to have an unimpaired medical status. All participants gave their written informed consent prior to inclusion. The study was approved by the local ethics committee and is registered in the German Register of Clinical Studies (DRKS00025791).

### Test material

Rey’s Auditory Verbal Learning Test (RAVLT), Wechsler Memory Scale IV (WMS), and Rey’s Complex Figure (RCF) were performed at baseline, 30 min, and 7 days. To maintain consistency across the study, we administered the tests in a predetermined sequence. First, the word list was presented followed by the presentation of the WMS stories. Lastly, Rey’s complex figure was shown. After this, the 30 min re-test was performed for the tests in the same sequence as the first presentation.

The logical verbal memory was investigated with the WMS story subtest where two stories of 25 items each were read. At the mentioned timepoints, participants had to recall as many items as possible. It is noteworthy, that the participants were not aware of the fact, that the learned items have to be recalled after 7 days. We, therefore, assumed a surprise effect in the 7 day testing. The primary outcome was defined as the ratio of the 7-day score divided by the 30 min score, expressed as *Q*_RAVLT_, *Q*_WMS_, or *Q*_RCF,_ respectively [11]. Participants underwent a neuropsychological testing and neuropsychiatric questionnaire using the Montreal Cognitive Assessment (MoCA), the Symbol Digit Modalities Test (SDMT), Epworth Sleepiness Scale (ESS), Fatigue Impact Scale (FIS), Pittsburgh Sleep Quality Index (PSQI), and Beck Depression Inventory II (BDI-II) [14]. To assess subjectively perceived memory function, a questionnaire for recording everyday memory experience (FEAG, higher scores indicate more forgetfulness in everyday life), the Everyday Memory Questionnaire (EMQ-S, higher scores indicate more forgetfulness in everyday life) and the questionnaire of Experienced Attention and Memory Deficits in Everyday Life (FEDA, higher scores indicate less attention and memory deficits) were used [15–17]. Several methods are described for recording an SMI. The present study used two established methods, firstly whether a SMI was noticed by the study participants and furthermore, a quantitative question about the extent of an SMI [5]. SMI was assessed in two ways: (i) by asking if participants felt more forgetful and (ii) by asking the participants to rate their forgetfulness on a scale from 0 (no complains) to 100 (severe memory deficit).

### Statistics

The Mann–Whitney-*U*-Test was used to analyse questionnaires and ALF-tests. Demographical data were analysed using chi^2^-test and the Fisher’s-Exact-test. The general linear regression model was built with group as the interaction variable, as the independent variable, we defined the subjective forgetfulness and FIS. *Q*_RAVLT/WMS_ were entered as the depended variable. We used Bonferroni correction for primary outcomes and, therefore, defined the significant *p*-value at 0.016 according to three main outcome measures (*Q*_RAVLT_, *Q*_WMS_ or *Q*_RCF_), SPSS was used for all statistical tests.

## Results

Thirty patients with relapsing–remitting MS and 30 HC matched for age and educational level were enrolled. Demographic variables are shown in Table 1. The mean EDSS of 1.06 (SD = 0.9) and the mean time since diagnosis of 2.7 years (SD = 3.06) indicate mildly affected MS patients.

**Table 1:** Baseline and clinical characteristics and neuropsychological results

	MS group	Controls	*p*-Value
Mean age (SD)	28.56 (3.83)	29.3 (6,38)	*p* = 0.900 (MWU)
Gender (m/f)	4/26	7/23	*p* = 0.317 (chi^2^-test)
Mean length of education (SD)	12.03 (0.96)	12.03 (1.24)	*p* = 0.676 (MWU)
School leaving certificate			*p* = 0.360 (Fisher Exact)
Middle school	1	–	
Junior Highschool	8	5	
A-levels	21	25	
SMI	7	1	*p* = 0.058 (chi^2^-test)
Forgetfulness	35.67	24.67	*p* = 0.004 (MWU)
EDSS mean (min/max; SD)	1.06 (0–3; 0.9)		
Time since initial diagnosis in years (min/max; SD)	2.73 (0–12; 3.06)		
Time since first manifestation in years (min/max; SD)	3.15 (0–12; 3.15)		
MS medication	21/30		
Relative result after 7 days			
RAVLT 7 days	51.11% (13–87)	64.89% (27–87)	p < 0.001
WMS 7 days	41.87% (18–69)	48% (26–70)	0.014
RCF 7 days	56.9% (18–89)	55.05% (7–92)	0.646
Relative result after 30 min			
RAVLT 30 min	76% (47–100)	79.11% (40–100)	0.258
WMS 30 min	48.27% (18–74)	48.53% (22–78)	0.953
RCF 30 min	66.85% (36–97)	62.1% (15–100)	0.300
Learning outcomes			
RAVLT (sum of trials I–V)	89.78% (67–100)	88.22% (67–100)	0.331
WMS (sum of trials I–V)	55.33% (28–74)	54.07% (22–80)	0.524
RCF (drawing from template)	100% (100–100)	99.86% (96–100)	0.317
PSQI (SD)	5.8 (0,5)	4.36(2,4)	*p* = 0.690
FIS-D (SD)	36.3 (21,2)	20.1 (15,8)	p < 0.001
ESS (SD)	6.5 (3,6)	5.6 (2,6)	*p* = 0.536
EMQ-S (SD)	26.4 (16,4)	17.3 (11,1)	*p* = 0.019
FEDA (SD)	110.9 (14,3)	120.5 (9,6)	*p* = 0.003
FEAG (SD)	86.3 (17,4)	76.0 (12,7)	*p* = 0.030
MoCA (SD)	28.9 (1,2)	28.8 (1,6)	*p* = 0.827
SDMT (SD)	61.1 (9,1)	59.4 (9,9)	*p* = 0.468
BDI (SD)	6.7 (4,6)	4.4 (3,7)	*p* = 0.460

*MS* multiple sclerosis; *EDSS* Expanded Disability Status Scale; *RAVLT* Rey’s Auditory Verbal Learning Test; *WMS* Wechsler Memory Scale; *RCF* Rey’s Complex Figures; *MWU test* Mann–Whitney *U* test; *SD* standard deviation; *m* male; *w* female; *min* minimum; *max* maximum; *PSQI* Pittsburg Sleep Quality Index; *FIS* Fatigue Impact Scale; *ESS* Epworth Sleepiness Scale; *EMQ-S* Everyday Memory Questionnaire; *FEDA* Questionnaire of Experienced Deficits of Attention; *FEAG* Everyday Memory Assessment Questionnaire; *MoCA* Montreal Cognitive Assessment; *BDI* Becks Depression Inventory II

Seven MS patients and one HC reported SMI when asked a dichotomic question (yes/no). However, when asked to rate their subjective everyday forgetfulness, the value was significantly greater in MS patients (mean 35.67 (SD = 15.9) vs 24.67 (SD = 9.37; *p* = 0.004) for HC. Initial learning and 30-min recall did not differ, as shown by the percentages of items remembered (Table 1).

**Fig. 1:**
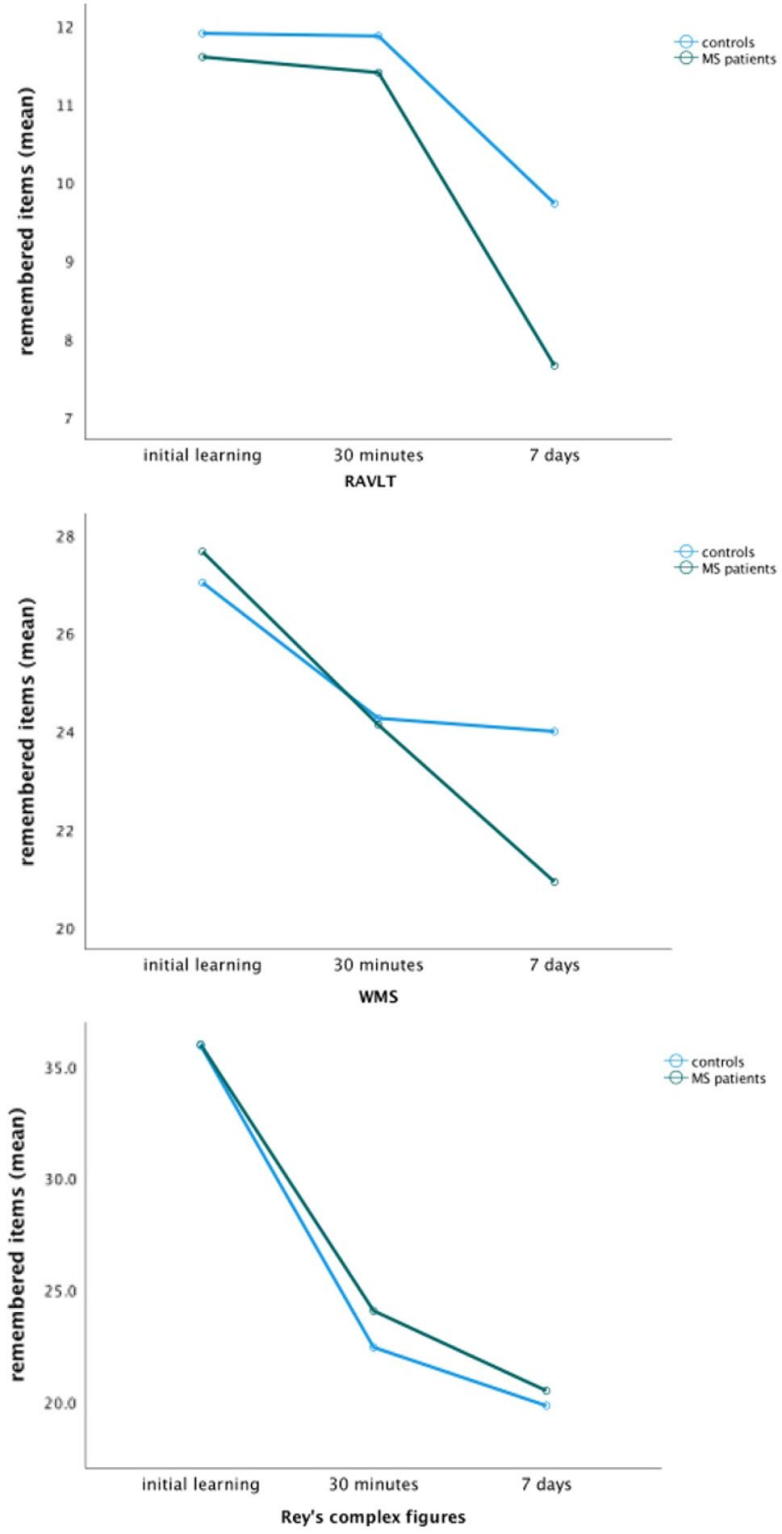
Mean results of the different time point for each subtest. *RAVLT* Rey’s Auditory Verbal Learning Test; *WMS* Wechsler Memory Scale; *RCF* Rey’s Complex Figures; *MS* multiple sclerosis

However, the *Q*_RAVLT_ was 0.66 (SD = 0.13) in the MS group vs 0.82 (SD = 0.16) in the HC group. Thus, significantly fewer items were recalled in the MS group (*p* < 0.016). *Q*_WMS_ of the MS-group was 0.88 (SD = 0.15) vs. 1.01 (*p* = 0.02) for healthy controls, showing a trend towards accelerated long-term forgetting for the story recall in the MS-group. The *Q*_RCF_ for the MS group was similar in both groups (0.84, SD = 0.22 vs. 0.88, SD = 0.17). In summary, there were significant results for the ALF score for verbal memory (Fig. 1). The result of the 30 min tests relative to the maximum score of the subtests was examined to investigate hippocampal forgetting. For the free recall of the word list, the MS group reached 76% (vs. 79.11% controls; *p* = 0.258). For the story learning, the patients achieved 48.27% (vs. 48.53%; *p* = 0.953). Again, the comparison regarding the geometric figure did not yield significant differences (MS 66.85% vs. 62.31%; *p* = 0.331). To assess the learning performance, the results of the last learning trial as percentages were compared. Here, no significant differences were found (MS 89.78% vs. 88.22%; *p* = 0.331). In addition, the ALF test score at 7 days for each subtest was compared in relation to the maximum score achievable. Patients remembered on average 51.11% of the word list, compared to 65.89% in the control group (*p* = 0.001), thus showing a significant difference as well. In free recall for the logical memory, the patients recalled an average of 41.87% of the items, while the control group came up with 48% after 7 days (*p* = 0.014). There was no difference for the complex figure after 7 days (MS 56.9% vs. 55.05%; *p* = 0.646).

Patients scored higher on the FIS, indicating greater fatigue. Some participants showed an impaired sleep quality when the cut-off was used. We used the chi2 test to examine those participants and their distribution across groups. This did not reveal any significant differences between the control and the MS group (chi2 = 1.067, *p* = 0.302). No significant results were found between groups for the PSQI, proposing an equal sleep quality in both groups. Patients reported significantly more forgetfulness on the EMQ, FEDA and FEAG. Subjective forgetfulness was greater in MS patients (mean 35.67 (SD = 15.9) vs. 24.67 (SD = 9.37; *p* = 0.004) in HC (Table 1). Neither group showed a pathological MoCA result (defined as a score lower than 26), or an impaired processing speed as measured with the SDMT.

In MS patients, the severity of subjective forgetfulness was significantly related to ALF performance (*Q*_RAVLT_) and to FIS scores indicating a negative relationship between subjectively experienced memory impairment and objectively measured ALF performance and fatigue severity, respectively (regression model Table 2). In other words, higher scores in the FIS or in the SMI rating resulted in a more pronounced ALF. No significant associations regarding fatigue or sleep disturbances were found in the regression model for the healthy control group.

**Table 2:** Results of the linear regression of the questionnaires with the primary outcome

Variable	Coefficient *B*	*p*-Value	95% CI
Subjective forgetfulness × *Q*_RAVLT_	*R*^2^ = 0.306		
MS group	− 0.004	0.01	−0.008 to −0.001
Control group	0.002	0.066	0.0–0.013
FIS × *Q*_RAVLT_	*R*^2^ = 0.294		
MS group	− 0.003	0.034	−0.005 to −0.00001
Control group	0.002	0.025	0.001–0.009
Subjective forgetfulness × FIS	*R*^2^ = 0.27		
MS group	0.0549	0.011	0.133–0.964
Control group	0.037	0.671	− 0.967 to 0.627

*CI* confidence interval; *FIS* Fatigue Impact Scale; *MS* multiple sclerosis

## Discussion

The present study demonstrates a significantly increased ALF of verbally encoded information in mildly affected MS patients whereas standard memory tests showed normal results in this group. ALF performance was directly related to SMI, as shown by regression analysis. Thus, ALF helps to quantify and objectify a subjective decline in everyday memory and transforming SMI into an objectively measurable entity of memory impairment. This finding may foster the acceptance, diagnostic vigilance, and therapeutic strategies to treat SMI.

By demonstrating a relatively brief duration since diagnosis or manifestation, together with test results that are comparable to, and in some cases even superior to those of the control group, and by presenting only non-pathological results in standard neuropsychiatric assessments, we conducted our study of Accelerated Long-Term Forgetting (ALF) within a cohort of patients mildly affected by MS. SMI could elude routine neuropsychological testing in the present study. One study reported a memory deficit in people with MS who reported a SMI in the first trail of a verbal memory task—which may reflect a real-world scenario in which information is only retrieved once [18]. In summary, prior research focused on the relationship between complaints of everyday memory deficits in individuals with Multiple Sclerosis (MS) and neuropsychological test results obtained in one session [19]. The present results add to this research by testing long-term memory retrieval using objective methods.

In other studies, subjects had to learn 10 semantically related words in a selected reminding technique [20]. The authors found intact recall performance after 1 week. Furthermore, Gaudino et al. found a higher number of learning attempts to the learning criterion in the MS group (7.1 vs. 5.1 repetitions) while there were no differences in recall after 1 week. Given semantically related words, we assume that features such as category learning or building cognitive strategies to bind this relation between words influence memory retrieval. We hypothesized that intact long-term memory for story recall may demonstrate a significant difference between people with MS and healthy controls in the present study, given the relation of information to be recalled. The visual learning and memory task (modified 7/24 visual memory test) used in the Gaudino study also differs from the RCFT used in our study. Hereby, the 7/24 repeatedly demonstrated dot combinations that must be learned while the Rey figure is displayed only once without the note that this figure has to be recalled at a later point in time. We interpret the differences between both studies by the mentioned methodological differences. The unimpaired recall of semantically related words in the Gaudino study might correspond to our results of intact story recall. ALF testing for the WMS revealed a trend for decreased long-term memory performance in the MS group. One possible explanation might be that the associative part of this memory task has helped to better memorize the items which in turn lead to better memory performances, even for participants which showed impaired results in the RAVLT. Contrary to verbal memory, no evidence of ALF was shown for visual memory. The groups did not differ significantly at any of the time points measured. Two explanations can be given for this finding. First, visual information is remembered significantly less well after 7 days, even in healthy subjects, which raises the question of whether the Rey figure test used is at all suitable for recording ALF, and secondly, Artemiadis et al. were able to show that only the EDSS correlates negatively with visuospatial memory [21]. Therefore, it seems conclusive that a patient population with such low EDSS as in the present study does not yet show limitations in this domain.

However, the present study showed that MS patients forgot significantly more information after 7 days. As learning trials and initial recall (30 min) showed no differences, we suggest that the findings of an accelerated long-term forgetting are better explained by a deficit in memory consolidation rather than memory encoding. Furthermore, patients’ everyday memory ratings are significantly related to ALF performance and subjective forgetting. This relationship may provide a practical approach to quantifying everyday memory deficits. However, future studies involving larger numbers of MS patients should confirm our findings and may be able to define thresholds for cut-off values for ALF performance to aid the diagnostic process of everyday memory decline in MS patients.

Long-term storage of information is based on sleep-dependent memory consolidation, but the group comparison showed similar sleep patterns for both groups. No significant results were found between groups for the PSQI, proposing an equal subjective sleep quality in both groups. Even, when a cut-off score for impaired sleep quality was used, both groups did not demonstrate significant differences. It can be concluded that subjective sleep quality cannot explain impaired ALF findings. Further studies should collect objective sleep parameters such as slow-wave sleep involved in memory consolidation [22]. However, we cannot rule out the possibility that changes in sleep architecture (e.g., a reduction in slow-wave-sleep) may interfere with sleep-dependent memory consolidation in our patient group. While some data suggest that the memory performance in MS is only to a little or no extent influenced by fatigue, the data of the present study indicate that in MS patients, fatigue severity is associated with subjective forgetfulness and ALF performance [23]. One potential mechanism could be the association of both via macro/microstructural and functional changes in brain activity. This could lead to alteration in the cortico-cortical and cortico-subcortical networks involved in the process of memory formation [24]. We acknowledge the fact, that ALF is a relatively new field in memory research and was mostly investigated in epilepsy patients. However, ALF got proven to be evident in various other conditions and a lot of research has been put into the challenge to overcome methodological issues, as described in the introduction [7–12] By strictly adhering to those methodological recommendations by Elliot et al. 2014, we tried to assured a valid assessment of ALF in MS. Accordingly, we matched patient and control groups rigorously, used both verbal and non-verbal test material and measured forgetting using both recall and recognition tests. Furthermore, we tried to avoid rehearsal and repeated recall. In addition, we took care that the immediate delay period was long enough to ensure information is stored in the long-term memory and retrieval is not reliant on short-term memory processes and matching of the initial learning. Future studies should (i) include a larger sample size to develop the ALF methodology as a diagnostic tool for SMI in MS, (ii) include structural and functional imaging revealing the mechanisms of ALF in MS, and (iii) assess whether ALF is a useful tool for directing the evolution of SMI towards an amnestic type of mild cognitive impairment. As cognitive and memory impairments in MS patients respond well to treatment options like specific learning strategies or computer-based interventions, early and objective quantification of SMI offers the potential to treat SMI in MS patients to prevent the negative impact of SMI on the quality of life in MS patients and to delay its progression to a mild cognitive impairment during the course of the disease [25, 26].

## Data Availability

The data that support the findings of this study are available from the corresponding author upon reasonable request.
